# The link between red blood cell distribution width and 3-month prognosis in acute ischemic stroke patients: a secondary analysis of a cohort study

**DOI:** 10.1186/s12883-025-04309-y

**Published:** 2025-07-30

**Authors:** Qin Xiong, Dong Kang, Shu-ming Xiong, Dong-ping Wu, Xin Luo, Wen-pei Zhang, Jing Tang, Zheng-guang He

**Affiliations:** 1 Department of Intensive Care Unit, The Third People’s Hospital of Suining, Suining, 629099 China; 2 Department of neurosurgery, The Third People’s Hospital of Suining, Suining, 629099 China; 3 Department of Respiratory Medicine, The Third People’s Hospital of Suining, Suining, 629099 China

**Keywords:** Ischemic stroke, Red blood cell distribution width, Poor prognosis, Modified Rankin scale

## Abstract

**Background:**

Few studies to date have explored correlations between red blood cell distribution width (RDW) and prognostic outcomes in acute ischemic stroke (AIS) patients, and conclusions based on the available evidence are controversial. This study was designed to better clarify the association between RDW and 3-month prognostic outcomes in AIS patients.

**Methods:**

This was a secondary analysis of a cohort study. The prospective cohort used to conduct these analyses was comprised of 1,901 AIS patients who underwent treatment in South Korea between January 2010 and December 2016. A modified Rankin Scale (mRS) value above 2 was used to define a poor 3-month prognosis. The link between RDW and poor 3-month prognostic outcomes in subjects with AIS was explored with a binary logistic regression model, with further subgroup analyses and interaction tests also being conducted.

**Results:**

Of 1,901 individuals analyzed in this study, 1,167 (61.4%) were male, and 543 (28.6%) met the criteria for poor 3-month outcomes. Following adjustment for confounding factors, RDW was positively correlated with poor 3-month outcomes in these individuals with AIS (OR = 1.12, 95% CI; 1.03–1.23, *P* = 0.0120). Subgroup analyses demonstrated a consistent positive association (OR > 1.0) between elevated RDW and adverse outcomes across the majority of baseline characteristics. No interactions were observed between RDW and poor 3-month outcomes among AIS patients.

**Conclusion:**

These results demonstrate a positive correlation between RDW and poor 3-month prognosis among AIS patients. The findings suggest RDW may serve as a practical prognostic biomarker, warranting further validation in diverse populations.

## Background

Acute ischemic stroke (AIS) is a leading global cause of mortality and disability, adversely impacting patients and societies as a whole [1, 2]. One of the major barriers to stroke management is the poor prognosis facing most patients, who face a risk of death, long-term disability, and significant reductions in quality of life [3, 4]. The modified Rankin score (mRS) is widely employed to assess evaluate prognostic outcomes in stroke patients, offering a tool that can be used to gauge limitations to the activities of daily life among affected individuals [5, 6]. Higher mRS scores are indicative of more severe disability and functional limitations [7]. Effective risk stratification, patient management, and targeted treatment development efforts hinge on the establishment of prognostic indicators that can be reliably implemented in clinical settings.

Red blood cell distribution width (RDW) can be utilized to measure peripheral red blood cell size variability, often being employed to evaluate patients for anemia [8]. RDW has been demonstrated to be linked to a higher risk of several forms of cardiovascular disease [9–11]. Relatively little is known about the association between RDW and the prognosis of patients with ischemic stroke, and the connection between the two remains somewhat controversial. A univariate analysis of 1,504 acute stroke patients revealed that RDW was associated with NIHSS scores (β = 0.24, *P* = 0.01) and poor functional outcomes (OR = 10.73, *P* < 0.001), though multivariate analysis did not confirm its independence [12]. Similarly, a study of 282 tissue plasminogen activator (tPA)-treated stroke patients stratified by RDW levels (< 12.9% vs. ≥13%) found no significant differences in 3-month modified Rankin Scale scores [13]. Conversely, a multicenter study of 847 first-time acute ischemic stroke patients demonstrated that each 1% increase in RDW raised the risk of poor functional outcomes by 22.2% (OR = 1.222, *P* = 0.006) and mortality by 39.5% (OR = 1.395, *P* < 0.001) [14]. Another study of 629 acute ischemic stroke patients, grouped by RDW quartiles, showed that higher RDW levels were significantly linked to moderate-to-severe stroke (OR = 2.21), poor 3-month functional outcomes (OR = 1.86), and lower Barthel Index scores (OR = 2.27) [15]. These findings conducted to date have been limited by their small sample sizes and lack of appropriate stratified analyses or sensitivity testing.

Higher RDW values are related to inflammatory activity, oxidative stress, and the impairment of erythropoiesis [16, 17]. As inflammation is closely tied to post-stroke pathology, RDW has the potential to impact stroke patient prognostic outcomes through its responsivity to the overall inflammatory state [18, 19]. We hypothesized that elevated RDW levels at hospital admission would be independently correlated with unfavorable functional outcomes (mRS > 2) at 3 months in patients with AIS. This study was devised to probe the link between RDW and poor 3-month mRS outcomes in individuals affected by AIS. The results of these analyses have the potential to inform the development of personalized therapeutic approaches and to improve patient outcomes.

## Methods

### Study design

This was a cohort study based on a previously conducted single-center prospective study performed in South Korea between January 2010 and December 2016 [20]. Here, RDW was utilized as an independent variable, while mRS scores for patients with AIS served as the dependent variable.

### Data source

Kang et al. [20] kindly made the original data used to conduct this study freely available. The findings were released through an unrestricted access model with a Creative Commons Attribution license, allowing for their unlimited sharing, distribution, and reproduction in any form so long as the initial source is properly acknowledged. We thank all parties who contributed the data used for this research effort.

### Study population

**Fig. 1:**
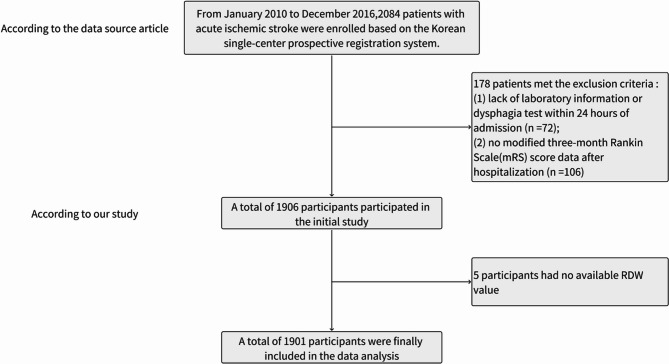
An overview of the selection process

The primary study included 2,084 individuals diagnosed with AIS, of whom 178 were excluded based on exclusion criteria established in the original study [20], including (1) the lack of any evaluation of dysphagia or laboratory testing conducted within 24 h of admission (*n* = 72), and (2) the lack of mRS scores calculated 3 months following hospital discharge (*n* = 106). The remaining 1,906 patients were retained in the initial study dataset. For the present secondary analysis, 5 additional patients without available RDW values were excluded. The patient selection process is outlined in Fig. 1.

AIS patients admitted to the hospital within 7 days of symptom onset were recruited by the original investigator team [20], using data from a single-center prospective registry [20]. The primary study was approved by Seoul National University Hospital’s institutional review board, which waived any requirement for informed patient consent (IRB No. 1009-062-332) [20]. No additional ethical oversight was deemed necessary for secondary analyses. The original study was performed as per the Declaration of Helsinki in accordance with appropriate laws and guidelines, and the same was true for this secondary analysis.

### Variables

RDW, which was measured within 24 h of admission and obtained from electronic medical records [20], was treated as a continuous variable.

### Patient 3-month prognostic outcomes

Patient outcomes at 3 months after AIS onset were analyzed based on mRS scores [21]. Follow-up information was collected via outpatient visits or telephone calls [20]. Patients were classified into those with a good prognosis (mRS ≤ 2) and those with a poor prognosis (mRS ≥ 3) [22].

### Covariates

All information related to data collection was derived from the original study [20]. Covariate selection was performed as per prior studies and our clinical experience. The covariates included in this study were categorized into continuous and categorical variables. The continuous variables comprised red blood cell (RBC) count, white blood cell (WBC) count, erythrocyte sedimentation rate (ESR), platelet count (PLT), triglycerides (TG), total cholesterol (TC), low-density lipoproteins (LDL), high-density lipoproteins (HDL), creatinine (CR), albumin (ALB), blood urea nitrogen (BUN), high sensitivity c-reactive protein (hs-CRP), body mass index (BMI), activated partial thromboplastin time (APTT), international normalized ratio (INR), glycosylated hemoglobin (HbA1c), and National Institutes of Health Stroke Scale (NIHSS) scores. The categorical variables included age, gender, hypertension, previous stroke or transient ischemic attack (TIA), dyslipidemia, coronary heart disease, diabetes, current smoking status, atrial fibrillation, and stroke etiology.

### Statistical analysis

Categorical data are given as frequencies and percentages, while continuous variables are given as the mean (standard deviation) or median (minimum and maximum). Categorical variables were compared with the χ2 test, while skewed and normally distributed continuous variables were compared with Kruskal-Wallis H tests and one-way ANOVAs. Initially, differences between RDW tertiles were analyzed [23], after which the impact of each variable on unfavorable AIS patient outcomes was assessed with a univariate logistic regression approach. Links between RDW and poor 3-month prognostic outcomes in AIS patients were then analyzed with three multivariate binary logistic regression models. Model 1 received no adjustment. Model 2 adjusted for age and gender. Model 3 was further adjusted for age, gender, BMI, current smoking, WBC, RBC, ESR, TC, TG, HDL, LDL, BUN, ALB, hs-CRP, INR, initial NIHSS score, previous stroke/TIA, hypertension, diabetes, hyperlipidemia, atrial fibrillation, and stroke etiology. Confounding factors were assessed by evaluating the impact of covariates on the effect estimate of RDW exceeding 10%, or the regression coefficient of covariates on poor prognosis at 3 months with a significance level of *P* < 0.1 [23, 24]. Sensitivity analyses were then conducted to assess result reliability. RDW values were converted into tertiles as a categorical variable, and the *P*-value for trend was calculated to validate results for RDW as a continuous variable, and to explore any possibility of nonlinearity. Subgroup analyses were performed using stratified binary logistic regression models to assess the association between RDW and poor prognosis across prespecified strata defined by age, gender, previous stroke/TIA, hypertension, diabetes, hyperlipidemia, current smoking, atrial fibrillation, coronary heart disease, and stroke etiology. Subgroup interactions were examined with a likelihood ratio test. Data was missing only for WBC (*n* = 1, 0.05%) and TG (*n* = 9, 0.47%). Given the negligible missingness, we implemented complete-case analysis without imputation [25]. EmpowerStats (X&Y Solutions, Inc., MA) and R (http://www.r-project.org) were used for statistical analyses, and a two-sided *P* < 0.05 was regarded as significant.

## Results

### Participant characteristics

Detailed participant demographic and clinical characteristics are presented in Table 1. This study enrolled 1,901 patients of whom 61.4% were male. This included 543 (28.6%) patients who exhibited a poor 3-month prognosis. The overall patient cohort included 435 (22.88%), 505 (26.56%), 668 (35.14%), and 293 (15.41%) who were respectively < 60, 60–70, 70–80, and > 80 years old. RDW values were used to separate patients into three tertiles, with the groups exhibiting low, medium, and high RDW values respectively being classified as those in the 11.10 − 12.60%, 12.70 − 13.40%, and 13.50 − 22.50% ranges.

BMI showed a decreasing trend with increasing RDW (*P* < 0.001). Laboratory indices indicated that RBC, PLT, TC, TG, HDL, and LDL were significantly lower in the high RDW tertile than in the low RDW tertile (all *P* < 0.05), while BUN, CR, and hs-CRP were significantly higher (all *P* < 0.001). Regarding clinical comorbidities, the prevalence of atrial fibrillation (29.07%) and coronary heart disease (14.85%) in the high RDW tertile was significantly higher than that in the low RDW tertile (16.43% and 8.75%, respectively; all *P* < 0.001). The mRS showed a higher proportion of moderate-to-severe disability (mRS 3–6) in the high RDW tertile (36.18%) compared to the low RDW tertile (24.46%, *P* < 0.001). Stroke etiologies differed significantly, with a higher proportion of CE (31.44%) and lower proportions of SVO and LAA in the high RDW tertile (all *P* < 0.001). No significant differences were observed in gender, WBC, HbA1c, APTT, current smoking, previous stroke/TIA, hypertension, diabetes, or hyperlipidemia across the three tertiles (all *P* > 0.05) (Table 1).

**Table 1:** The basic traits of participants

RDW tertiles, %	Low(11.10–12.60)	Middle(12.70–13.40)	High(13.50–22.50)	*P*-value
No. of subjects	560	708	633	
Age, n (%)				< 0.001
< 60	166 (29.64%)	166 (23.45%)	103 (16.27%)	
60 to < 70	159 (28.39%)	194 (27.40%)	152 (24.01%)	
70 to < 80	169 (30.18%)	242 (34.18%)	257 (40.60%)	
≥ 80	66 (11.79%)	106 (14.97%)	121 (19.12%)	
Gender, n (%)				0.495
Male	342 (61.07%)	446 (62.99%)	379 (59.87%)	
Female	218 (38.93%)	262 (37.01%)	254 (40.13%)	
BMI, mean (SD), kg/m^2^	23.98 (3.20)	23.78 (3.19)	22.78 (3.25)	< 0.001
WBC, mean (SD), 10^9^/L	8.32 (2.88)	8.07 (2.64)	8.05 (3.15)	0.196
RBC, mean (SD), 10^12^/L	4.47 (0.53)	4.39 (0.58)	4.12 (0.73)	< 0.001
PLT, mean (SD), 10^9^/L	229.62 (57.62)	224.30 (68.46)	217.34 (83.90)	0.011
ESR, median (min-max), mm/h	0.00 (0.00-140.00)	0.00 (0.00-107.00)	0.00 (0.00-138.00)	< 0.001
TC, mean (SD), mg/dL	187.44 (43.35)	180.04 (41.53)	171.65 (45.61)	< 0.001
TG, mean (SD), mg/dL	111.05 (65.75)	105.10 (57.14)	100.92 (57.45)	0.014
HDL, mean (SD), mg/dL	43.80 (16.44)	45.77 (16.31)	42.66 (17.52)	0.003
LDL, mean (SD), mg/dL	108.58 (42.59)	104.84 (41.39)	99.89 (42.88)	0.002
BUN, mean (SD), mg/dL	16.22 (6.93)	17.06 (7.17)	19.41 (11.48)	< 0.001
CR, median (min-max), mg/dL	0.86 (0.37–11.12)	0.90 (0.36–13.91)	0.91 (0.37–11.38)	< 0.001
ALB, mean (SD), g/dL	4.18 (0.35)	4.06 (0.36)	3.84 (0.49)	< 0.001
HbA1c, mean (SD), %	5.00 (2.94)	5.14 (2.73)	5.14 (2.61)	0.596
hs-CRP, median (min-max), mg/dL	0.10 (0.00-22.61)	0.12 (0.00-28.06)	0.19 (0.00-36.80)	< 0.001
INR, median (min-max)	1.01 (0.24)	1.04 (0.36)	1.07 (0.35)	0.007
APTT, median (min-max), s	31.11 (6.46)	30.58 (5.32)	30.77 (7.66)	0.353
Initial NIHSS score, median (min-max)	3.00 (0.00–25.00)	3.00 (0.00–33.00)	4.00 (0.00–28.00)	< 0.001
Current smoking, n (%)				0.547
No	341 (60.89%)	418 (59.04%)	392 (61.93%)	
Yes	219 (39.11%)	290 (40.96%)	241 (38.07%)	
Previous stroke/TIA, n (%)				0.126
No	456 (81.43%)	560 (79.10%)	485 (76.62%)	
Yes	104 (18.57%)	148 (20.90%)	148 (23.38%)	
Hypertension, n (%)				0.900
No	208 (37.14%)	258 (36.44%)	227 (35.86%)	
Yes	352 (62.86%)	450 (63.56%)	406 (64.14%)	
Diabetes, n (%)				0.568
No	380 (67.86%)	489 (69.07%)	420 (66.35%)	
Yes	180 (32.14%)	219 (30.93%)	213 (33.65%)	
Hyperlipidemia, n (%)				0.091
No	342 (61.07%)	439 (62.01%)	422 (66.67%)	
Yes	218 (38.93%)	269 (37.99%)	211 (33.33%)	
Atrial fibrillation, n (%)				< 0.001
No	468 (83.57%)	580 (81.92%)	449 (70.93%)	
Yes	92 (16.43%)	128 (18.08%)	184 (29.07%)	
Coronary heart disease, n (%)				0.003
No	511 (91.25%)	631 (89.12%)	539 (85.15%)	
Yes	49 (8.75%)	77 (10.88%)	94 (14.85%)	
mRS score, n (%)				< 0.001
0–2	423 (75.54%)	531 (75.00%)	404 (63.82%)	
3–6	137 (24.46%)	177 (25.00%)	229 (36.18%)	
Stroke etiology, n (%)				< 0.001
SVO	193 (34.46%)	240 (33.90%)	174 (27.49%)	
LAA	118 (21.07%)	154 (21.75%)	93 (14.69%)	
CE	123 (21.96%)	167 (23.59%)	199 (31.44%)	
Other determined	43 (7.68%)	45 (6.36%)	82 (12.95%)	
Undetermined	83 (14.82%)	102 (14.41%)	85 (13.43%)	

*BMI* body mass index, *WBC* white blood cell, *RBC* red blood cell, *PLT* platelet count, *ESR* erythrocyte Sedimentation Rate, *TC* total cholesterol, *TG* triglycerides, *HDL* high-density lipoproteins, *LDL* low-density lipoproteins, *BUN* blood Urea Nitrogen, *CR* creatinine, *ALB* albumin, *HbA1c* glycosylated hemoglobin, *hs-CRP* high sensitive c-reactive protein, *INR* international normalized ratio, *APTT* activated partial thromboplastin time, *NIHSS* National Institutes of Health Stroke Scale, *TIA* transient ischemia attack, *mRS* modified rankin scale, *SVO* small-vessel occlusion, *LAA* large-artery atherosclerosis, *CE* cardioembolism

### Univariate analysis

As reflected in Table 2, univariate analysis identified distinct associations between clinical variables and 3-month poor prognosis after AIS. Positive correlations were observed in RDW (OR = 1.26, *P* < 0.001), advanced age (70–80 years: OR = 1.89, ≥ 80 years: OR = 3.93, both *P* < 0.001), female sex (OR = 1.66, *P* < 0.001), inflammatory markers [WBC (OR = 1.08), ESR (OR = 1.02), hs-CRP (OR = 1.15), all *P* < 0.001], renal index (BUN: OR = 1.02, *P* = 0.004), coagulation marker (INR: OR = 1.43, *P* = 0.017), and acute stroke severity (NIHSS score: OR = 1.23, *P* < 0.001). Comorbidities including prior stroke/TIA (OR = 1.81), hypertension (OR = 1.34), diabetes (OR = 1.43), atrial fibrillation (OR = 1.99, all *P* < 0.01), and stroke etiologies of CE (OR = 1.48) and other determined causes (OR = 2.03, both *P* < 0.01) also showed positive associations. Conversely, negative correlations were found in TC (OR = 1.00, *P* = 0.0002), TG (OR = 1.00, *P* < 0.0001), LDL (OR = 1.00, *P* = 0.0014), higher BMI (OR = 0.92, *P* < 0.001), RBC (OR = 0.59, *P* < 0.001), ALB (OR = 0.28, *P* < 0.001), HDL (OR = 0.99, *P* = 0.03), current smoking (OR = 0.61, *P* < 0.001), hyperlipidemia (OR = 0.79, *P* = 0.02), and LAA (OR = 0.62, *P* = 0.003). Variables without significant associations included PLT, CR, HbA1c, APTT, and coronary heart disease (all *P* > 0.05).

**Table 2:** Univariate analysis of poor prognosis at 3 months after acute ischemic stroke

	Statistics	Odds ratio (95% CI)	*P*-value
Age, n (%)			
< 60	435 (22.88%)	1.0	
60 to < 70	505 (26.56%)	1.15 (0.84, 1.58)	0.3897
70 to < 80	668 (35.14%)	1.89 (1.42, 2.52)	< 0.0001
≥ 80	293 (15.41%)	3.93 (2.82, 5.47)	< 0.0001
Gender, n (%)			
Male	1167 (61.39%)	1.0	
Female	734 (38.61%)	1.66 (1.36, 2.03)	< 0.0001
BMI, mean (SD), kg/m^2^	23.50 ± 3.25	0.92 (0.89, 0.95)	< 0.0001
WBC, mean (SD), 10^9^/L	8.14 ± 2.89	1.08 (1.04, 1.12)	< 0.0001
RBC, mean (SD), 10^12^/L	4.33 ± 0.64	0.59 (0.50, 0.69)	< 0.0001
PLT, mean (SD), 10^9^/L	223.55 ± 71.31	1.00 (1.00, 1.00)	0.2929
ESR, median (min-max), mm/h	0.00 (0.00-140.00)	1.02 (1.01, 1.02)	< 0.0001
TC, mean (SD), mg/dL	179.43 ± 43.88	1.00 (0.99, 1.00)	0.0002
TG, mean (SD), mg/dL	105.45 ± 60.00	1.00 (0.99, 1.00)	< 0.0001
HDL, mean (SD), mg/dL	44.15 ± 16.81	0.99 (0.99, 1.00)	0.0295
LDL, mean (SD), mg/dL	104.29 ± 42.37	1.00 (0.99, 1.00)	0.0014
BUN, mean (SD), mg/dL	17.59 ± 8.88	1.02 (1.01, 1.03)	0.0037
CR, median (min-max), mg/dL	0.89 (0.36–13.91)	1.01 (0.92, 1.11)	0.7602
ALB, mean (SD), g/dL	4.02 ± 0.43	0.28 (0.22, 0.35)	< 0.0001
HbA1c, mean (SD), %	5.10 ± 2.75	1.01 (0.97, 1.04)	0.6898
hs-CRP, median (min-max), mg/dL	0.12 (0.00-36.80)	1.15 (1.11, 1.20)	< 0.0001
INR, median (min-max)	1.04 ± 0.33	1.43 (1.07, 1.92)	0.0167
APTT, median (min-max), s	30.80 ± 6.51	0.99 (0.98, 1.01)	0.2914
Initial NIHSS score, median (min-max)	3.00 (0.00–33.00)	1.23 (1.20, 1.26)	< 0.0001
Current smoking, n (%)			
No	1151 (60.55%)	1.0	
Yes	750 (39.45%)	0.61 (0.50, 0.75)	< 0.0001
Previous stroke/TIA, n (%)			
No	1501 (78.96%)	1.0	
Yes	400 (21.04%)	1.81 (1.44, 2.29)	< 0.0001
Hypertension, n (%)			
No	693 (36.45%)	1.0	
Yes	1208 (63.55%)	1.34 (1.09, 1.66)	0.0063
Diabetes, n (%)			
No	1289 (67.81%)	1.0	
Yes	612 (32.19%)	1.43 (1.16, 1.77)	0.0007
Hyperlipidemia, n (%)			
No	1203 (63.28%)	1.0	
Yes	698 (36.72%)	0.79 (0.64, 0.97)	0.0245
Atrial fibrillation, n (%)			
No	1497 (78.75%)	1.0	
Yes	404 (21.25%)	1.99 (1.58, 2.50)	<0.0001
Coronary heart disease, n (%)			
No	1681 (88.43%)	1.0	
Yes	220 (11.57%)	1.03 (0.76, 1.40)	0.8540
Stroke etiology, n (%)			
SVO	607 (31.93%)	1.0	
LAA	365 (19.20%)	0.62 (0.45, 0.85)	0.0031
CE	489 (25.72%)	1.48 (1.14, 1.91)	0.0029
Other determined	170 (8.94%)	2.03 (1.43, 2.89)	< 0.0001
Undetermined	270 (14.20%)	0.86 (0.61, 1.19)	0.3598
RDW, mean (SD), %	13.36 ± 1.37	1.26 (1.17, 1.35)	< 0.0001

### Associations between RDW and poor 3-month prognostic outcomes in AIS patients

Under Model 1, every 1 unit increase in RDW was linked to a 26% rise in the risk of a poor 3-month prognosis (OR = 1.26, *P* < 0.0001). In Model 2, the results did not show obvious changes (OR = 1.24, *P* < 0.0001). Under Model 3, the association showed a slight attenuation while maintaining statistical significance. Specifically, each 1 unit increase in RDW was associated with a 12% elevated risk of poor 3-month prognosis (OR = 1.12, *P* = 0.0120). Analyses of RDW tertiles showed that compared with the low RDW group, the high RDW group had a 75% higher risk of poor prognosis in the unadjusted model (Model 1: OR = 1.75, 95%CI 1.36–2.25, *P* < 0.0001) and a 55% higher risk in the model adjusted for age and gender (Model 2: OR = 1.55, 95%CI 1.19–2.01, *P* = 0.001). However, this association was no longer significant in the fully adjusted model (Model 3: OR = 1.08, 95%CI 0.78–1.50, *P* = 0.6371). Trend analyses also revealed that under Model 1 (*P* < 0.0001) and Model 2 (*P* = 0.0005), increases in RDW levels exhibited a significant linear association with a risk of poor prognostic outcomes, but this trend was no longer significant under Model 3 (*P* = 0.5782) (Table 3).

**Table 3:** Relationship between RDW and poor prognosis at 3 months after AIS in different models

Exposure	Model 1 OR (95%CI) *P* value	Model 2 OR (95%CI) *P* value	Model 3 OR (95%CI) *P* value
RDW (per 1 unit increase)	1.26 (1.17, 1.35) < 0.0001	1.24 (1.15, 1.33) < 0.0001	1.12 (1.03, 1.23) 0.0120
RDW tertiles			
Low	Ref	Ref	Ref
Middle	1.03 (0.80, 1.33) 0.8263	0.97 (0.75, 1.26) 0.8262	0.85 (0.62, 1.16) 0.3142
High	1.75 (1.36, 2.25) < 0.0001	1.55 (1.19, 2.01) 0.0010	1.08 (0.78, 1.50) 0.6371
P for trend	1.35 (1.18, 1.53) < 0.0001	1.26 (1.11, 1.44) 0.0005	1.05 (0.89, 1.23) 0.5782

Model 1: Non-adjusted model

Model 2: Adjusted for age and gender

Model 3: Adjusted for age, gender, BMI, current smoking, WBC, RBC, ESR, TC, TG, HDL, LDL, BUN, ALB, hs-CRP, INR, initial NIHSS score, previous stroke/TIA, hypertension, diabetes, hyperlipidemia, atrial fibrillation, stroke etiology

### Subgroup analyses

**Fig. 2:**
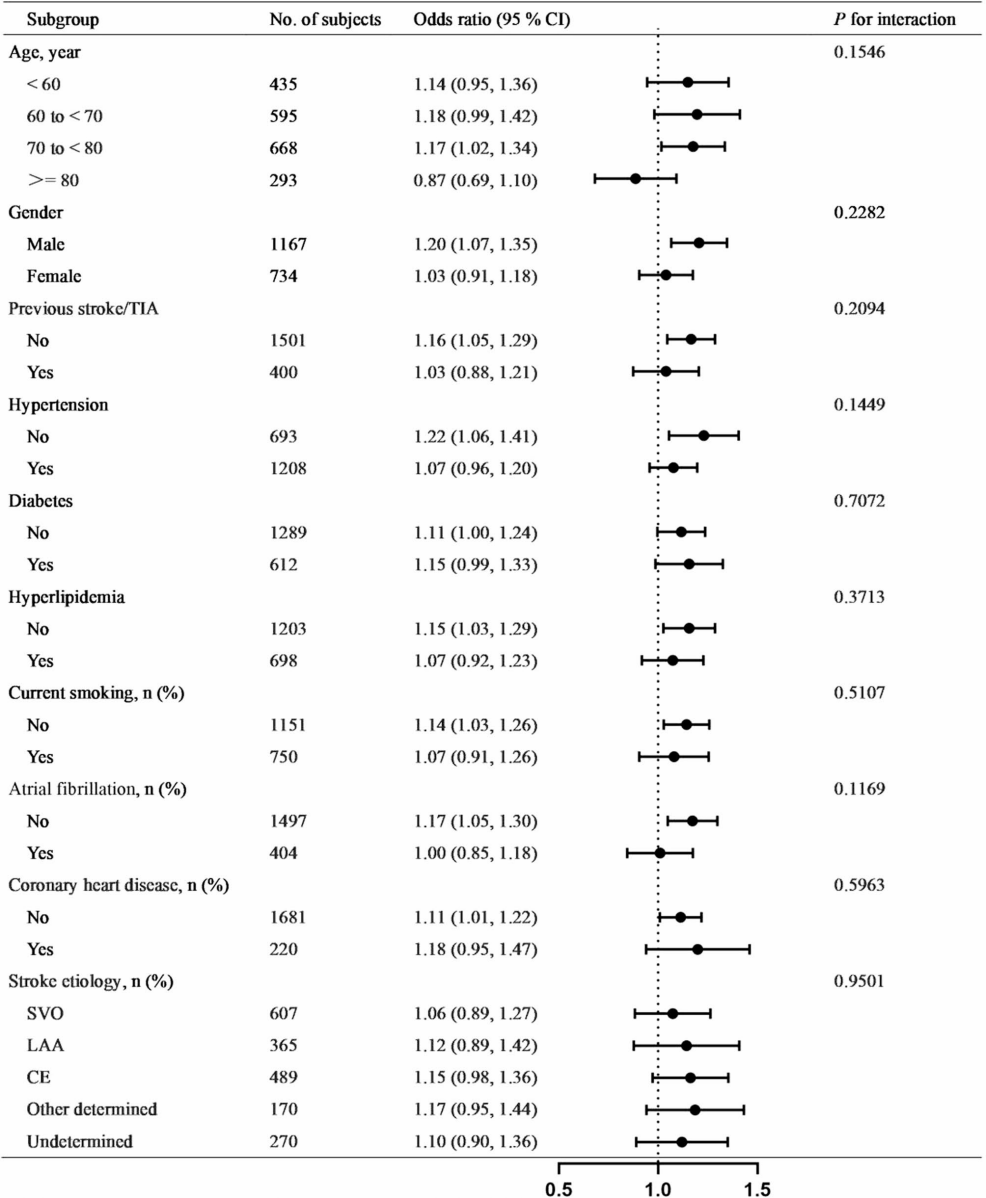
Relationships between RDW and poor prognosis at 3 months after AIS in various subgroups

Subgroup analyses revealed a consistently positive association between elevated RDW and poor 3-month outcomes across most predefined strata. Statistically significant associations (*P* < 0.05) were observed in males (OR 1.20, 95% CI 1.07–1.35), patients without prior stroke/TIA (OR 1.16, 1.05–1.29), non-hypertensive individuals (OR 1.22, 1.06–1.41), those without hyperlipidemia (OR 1.15, 1.03–1.29), non-smokers (OR 1.14, 1.03–1.26), patients without atrial fibrillation (OR 1.17, 1.05–1.30), and those without coronary heart disease (OR 1.11, 1.01–1.22). In contrast, a non-significant inverse trend was noted in the ≥ 80 years subgroup (OR 0.87, 0.69–1.10). No significant interactions were detected across all subgroups (P-interaction > 0.05) (Fig. 2).

## Discussion

This study was developed to probe the relationship between RDW and poor 3-month prognostic outcomes among patients with AIS. This is the largest such study conducted to date, and these analyses revealed a positive linear correlation between RDW and poor 3-month prognostic outcomes in AIS patients. This relationship remained intact following adjustment for possible confounding variables (OR = 1.12, 95% CI: 1.03–1.23, *P* = 0.0120), with a 12% increase in the risk of a poor 3-month prognosis being observed for each additional unit (1%) rise in RDW.

In past studies, the association between RDW and ischemic stroke patient prognosis has been subject to some degree of controversy. A retrospective hospital-based study of 847 consecutive first-episode AIS patients conducted a stepwise logistic regression analysis following adjustment for relevant confounding factors, revealing that higher RDW values were related to poor functional prognostic outcomes at 3 months (OR = 1.222,95% CI 1.059–1.409, *P* = 0.006) [26]. In a separate cohort study of 629 AIS patients, following multivariate logistic adjustment for confounding factors, a higher RDW was significantly related to higher mRS scores fo 3–6 after 3 months (OR 1.86, 95% CI, 1.02–3.41, *P* = 0.044) [15]. The meta-analysis additionally indicated that elevated RDW is associated with poor functional outcomes following stroke (OR/RR 1.220 at discharge; OR/RR 1.277 at 90 days) as well as an increased mortality rate (OR/RR 1.278) [27]. These findings have not been universal, however. For instance, in one retrospective analysis of 1,504 consecutive AIS patients, multivariate regression indicated that RDW as assessed on admission was not predictive of functional prognostic outcomes in stroke patients [12]. In a separate retrospective study of 570 AIS patients who underwent intravenous thrombolytic treatment, multivariate analyses suggested that RDW at baseline was not related to 1-year survival function among stroke survivors [28]. A meta-analysis also revealed that RDW is not significantly associated with poor functional outcomes at three months (mRS score ≥ 3) (OR 1.05, 95% CI 0.95–1.16) [29]. Several factors may account for these discrepant results, including sample size, differences in population characteristics (age, ethnicity, gender, underlying comorbidities, stroke severity, etc.), the chosen time points for RDW measurement, and/or differences in the confounding factors adjusted for in these studies. Most of these prior analyses included relatively small sample populations, were retrospective in nature, and did not incorporate sensitivity or subgroup analyses. The present study incorporated 1,901 AIS patients. Following adjustment for relevant confounding factors, baseline RDW in this patient population was positively correlated with a poor 3-month prognosis (OR = 1.12, 95% CI: 1.03–1.23, *P* = 0.0120).

The specific mechanistic basis underlying the association between RDW and poor prognostic outcomes after AIS remains elusive. Several potential mechanisms have been proposed in previous investigations, which can be categorized into the following aspects:

1. Inflammatory response: Inflammation is a key contributor to poor stroke outcomes. Elevated RDW values have been consistently associated with heightened systemic inflammation [30, 31]. During the post-stroke inflammatory cascade, cytokines such as interleukin-6 (IL-6) and tumor necrosis factor-α (TNF-α) are released, promoting oxidative stress and increasing erythrocyte membrane permeability [32]. This oxidative damage causes heterogeneous RBC maturation, leading to increased RDW as a marker of RBC size variability [33].
2. Oxidative stress: RDW elevation may reflect oxidative stress-induced RBC dysregulation. Oxidative stress not only disrupts normal RBC production but also impairs vascular endothelial function, promotes thrombosis, and exacerbates ischemic injury [34–37]. These effects collectively contribute to worse clinical outcomes after AIS, suggesting a bidirectional relationship between RDW, oxidative stress, and stroke prognosis.
3. Hemorheological alterations: Increased RDW is often accompanied by elevated blood viscosity and reduced RBC deformability, which can impede microcirculatory perfusion [38, 39]. In the context of stroke, these hemorheological changes may further compromise cerebral blood flow, leading to extended ischemic damage and poorer recovery.
4. Nutritional status: Low-grade malnutrition, particularly deficiencies in vitamins essential for RBC maturation (e.g., folate, vitamin B12), has been linked to higher RDW values [40]. Such nutritional deficiencies can impair neurovascular repair mechanisms, potentially contributing to adverse post-stroke outcomes.

Collectively, RDW likely influences post-stroke neural recovery through interconnected pathways of inflammation, oxidative stress, hemorheological dysfunction, and nutritional status. Longitudinal studies integrating mechanistic analyses are needed to clarify whether RDW represents a modifiable therapeutic target or merely a biomarker of systemic stress.

To assess the robustness of the association between the RDW and 3-month poor functional outcomes and to explore potential effect modification, we conducted pre-specified subgroup analyses across several clinically relevant baseline characteristics. The subgroup analyses demonstrated consistent directionality of the RDW-outcome association (OR > 1.0) across most baseline characteristics, with nonsignificant interaction *P*-values supporting robustness. Interestingly, the point estimate in the ≥ 80 years subgroup suggested a potential inverse association (OR = 0.87, 95% CI: 0.69–1.10; P-interaction = 0.1546). However, given the non-significant interaction term and the relatively small sample size within this stratum, this finding warrants cautious interpretation and further investigation.

Key strengths of this study include the fact that this is the largest sample size used to probe the link between RDW and poor prognostic outcomes in AIS patients to date. Additionally, the association between RDW and poor prognosis was explored using strict statistical adjustments in an effort to ensure clarity, while sensitivity and subgroup analyses were conducted to ensure the results were reliable.

This study is subject to certain limitations. First, as an observational and retrospective analysis, the study design may introduce selection bias and inherently limits the ability to establish causal relationships between exposures and outcomes. For one, these analyses were performed in Korean patient cohort such that validation in other ethnic groups will be necessary. In addition, age was treated as a categorical rather than a continuous variable in the original study such that the available data may be incomplete. Moreover, RDW was only measured a single time on admission such that it was not possible to assess any relationships between dynamic RDW changes and poor post-stroke outcomes. Lastly, this was a secondary analysis of a published dataset, and the original data did not include details related to potentially relevant confounding factors including a detailed medication history or intravenous thrombolysis.

## Conclusion

In this Korean cohort of AIS patients, elevated RDW demonstrates a modest but consistent association with poor functional outcomes. While categorical analyses showed attenuated significance upon comprehensive adjustment, the persistent directional risk pattern supports RDW’s potential clinical relevance in this population. Further validation in larger cohorts is warranted, particularly exploring age-related variations.

## Data Availability

The “PLoS One” database allows for the downloading of data (https://journals.plos.org/plosone/article?id=https://doi.org/10.1371/journal.pone.0228738IF: 2.9 Q1 B3).

## References

[1] Wang G, Zhang Z, Ayala C, Dunet DO, Fang J, George MG. Costs of hospitalization for stroke patients aged 18–64 years in the united States. J Stroke Cerebrovasc. 2014;23(5):861–8. 10.1016/j.jstrokecerebrovasdis.2013.07.017 PMC4544732 23954598

[2] Abedi V, Avula V, Razavi SM, Bavishi S, Chaudhary D, Shahjouei S, et al. Predicting short and long-term mortality after acute ischemic stroke using EHR. J Neurol Sci. 2021;427:117560. 34218182 10.1016/j.jns.2021.117560 PMC8480306

[3] Nagayoshi M, Everson-Rose SA, Iso H, Mosley TJ, Rose KM, Lutsey PL. Social network, social support, and risk of incident stroke: atherosclerosis risk in communities study. Stroke. 2014;45(10):2868–73. 25139878 10.1161/STROKEAHA.114.005815 PMC4201236

[4] Tang S, Yin J, Liu C, Sun M, Lee J, Sun Y, et al. Low pulse pressure after acute ischemic stroke is associated with unfavorable outcomes: the Taiwan stroke registry. J Am Heart Assoc. 2017;6(6):e005113. 28642220 10.1161/JAHA.116.005113 PMC5669158

[5] Broderick JP, Adeoye O, Elm J. Evolution of the modified Rankin scale and its use in future stroke trials. Stroke. 2017;48(7):2007–12. 28626052 10.1161/STROKEAHA.117.017866 PMC5552200

[6] Banks JL, Marotta CA. Outcomes validity and reliability of the modified Rankin scale: implications for stroke clinical trials: a literature review and synthesis. Stroke. 2007;38(3):1091–6. 17272767 10.1161/01.STR.0000258355.23810.c6

[7] Leppert MH, Poisson SN, Carroll JD, Thaler DE, Kim CH, Orjuela KD, et al. Cost-Effectiveness of patent foramen ovale closure versus medical therapy for secondary stroke prevention. Stroke. 2018;49(6):1443–50. 29720435 10.1161/STROKEAHA.117.020322 PMC5970986

[8] Elbayiyev S, Simsek GK, Ceran B, Akin MS, Kanmaz KH, Canpolat FE. Could red cell distribution width be used for predicting cardiac injury in neonates with COVID-19? J Med Virol. 2022;94(12):5739–45. 35938314 10.1002/jmv.28050 PMC9538182

[9] Amar D, Sinnott-Armstrong N, Ashley EA, Rivas MA. Graphical analysis for phenome-wide causal discovery in genotyped population-scale biobanks. Nat Commun. 2021;12(1):350. 33441555 10.1038/s41467-020-20516-2 PMC7806647

[10] Cheng X, Mell B, Alimadadi A, Galla S, Mccarthy CG, Chakraborty S, et al. Genetic predisposition for increased red blood cell distribution width is an early risk factor for cardiovascular and renal comorbidities. Dis Model Mech. 2020;13(5):dmm044081. 32238420 10.1242/dmm.044081 PMC7325433

[11] Horne BD, Anderson JL, Muhlestein JB, Ridker PM, Paynter NP. Complete blood count risk score and its components, including RDW, are associated with mortality in the JUPITER trial. Eur J Prev Cardiol. 2015;22(4):519–26. 24403296 10.1177/2047487313519347

[12] Ntaios G, Gurer O, Faouzi M, Aubert C, Michel P. Red cell distribution width does not predict stroke severity or functional outcome. Int J Stroke. 2012;7(1):2–6. 21645270 10.1111/j.1747-4949.2011.00609.x

[13] Shahsavarinia K, Ghavam LY, Moharramzadeh P, Pouraghaei M, Sadeghi-Hokmabadi E, Seifar F, et al. The predictive value of red cell distribution width for stroke severity and outcome. Bmc Res Notes. 2020;13(1):288. 32539809 10.1186/s13104-020-05125-y PMC7294627

[14] Kim DY, Hong DY, Kim SY, Park JJ, Kim JW, Park SO, et al. Prognostic value of red blood cell distribution width in predicting 3-month functional outcome of patients undergoing thrombolysis treatment for acute ischemic stroke. Medicine. 2021;100(37):e27255. 34664873 10.1097/MD.0000000000027255 PMC8447982

[15] Xue J, Zhang D, Zhang XG, Zhu XQ, Xu XS, Yue YH. Red cell distribution width is associated with stroke severity and unfavorable functional outcomes in ischemic stroke. Front Neurol. 2022;13:938515. 36438973 10.3389/fneur.2022.938515 PMC9682065

[16] Chen Q, Zhao B, Wang MY, Chen XY, Li D, Jiang XQ, et al. Associations between the red blood cell distribution width and primary angle-closure glaucoma: a potential for disease prediction. Epma J. 2019;10(2):185–93. 31258822 10.1007/s13167-019-00166-1 PMC6562012

[17] Hsueh CY, Lau HC, Li S, Tao L, Zhang M, Gong H, et al. Pretreatment level of red cell distribution width as a prognostic Indicator for survival in a large cohort study of male laryngeal squamous carcinoma. Front Oncol. 2019;9:271. 31041191 10.3389/fonc.2019.00271 PMC6477051

[18] Xiang YX, Wang WX, Xue Z, Zhu L, Wang SB, Sun ZH. Electrical stimulation of the vagus nerve protects against cerebral ischemic injury through an anti-infammatory mechanism. Neural Regen Res. 2015;10(4):576–82. 26170817 10.4103/1673-5374.155430 PMC4424749

[19] Hou P, Xue HP, Mao XE, Li YN, Wu LF, Liu YB. Inflammation markers are associated with frailty in elderly patients with coronary heart disease. Aging. 2018;10(10):2636–45. 30325739 10.18632/aging.101575 PMC6224228

[20] Kang MK, Kim TJ, Kim Y, Nam KW, Jeong HY, Kim SK, et al. Geriatric nutritional risk index predicts poor outcomes in patients with acute ischemic stroke - Automated undernutrition screen tool. PLoS ONE. 2020;15(2):e228738. 10.1371/journal.pone.0228738 PMC7017988 32053672

[21] Lord AS, Langefeld CD, Sekar P, Moomaw CJ, Badjatia N, Vashkevich A, et al. Infection after intracerebral hemorrhage: risk factors and association with outcomes in the ethnic/racial variations of intracerebral hemorrhage study. Stroke. 2014;45(12):3535–42. 25316275 10.1161/STROKEAHA.114.006435 PMC4245453

[22] Lv G, Wang GQ, Xia ZX, Wang HX, Liu N, Wei W, et al. Influences of blood lipids on the occurrence and prognosis of hemorrhagic transformation after acute cerebral infarction: a case-control study of 732 patients. Military Med Res. 2019;6(1):2. 10.1186/s40779-019-0191-z PMC6341695 30665465

[23] Ao T, Huang Y, Zhen P, Hu M. Association between red cell distribution width and 30-day mortality in patients with sepsis-associated liver injury: a retrospective cohort study. Front Med-Lausanne. 2024;11:1510997. 39744524 10.3389/fmed.2024.1510997 PMC11688371

[24] Kernan WN, Viscoli CM, Brass LM, Broderick JP, Brott T, Feldmann E, et al. Phenylpropanolamine and the risk of hemorrhagic stroke. New Engl J Med. 2000;343(25):1826–32. 11117973 10.1056/NEJM200012213432501

[25] Jakobsen JC, Gluud C, Wetterslev J, Winkel P. When and how should multiple imputation be used for handling missing data in randomised clinical trials - a practical guide with flowcharts. Bmc Med Res Methodol. 2017;17(1):162. 29207961 10.1186/s12874-017-0442-1 PMC5717805

[26] Kim J, Kim YD, Song TJ, Park JH, Lee HS, Nam CM, et al. Red blood cell distribution width is associated with poor clinical outcome in acute cerebral infarction. Thromb Haemostasis. 2012;108(2):349–56. 22739700 10.1160/TH12-03-0165

[27] Song SY, Hua C, Dornbors DR, Kang RJ, Zhao XX, Du X, et al. Baseline red blood cell distribution width as a predictor of stroke occurrence and outcome: A comprehensive Meta-Analysis of 31 studies. Front Neurol. 2019;10:1237. 31849813 10.3389/fneur.2019.01237 PMC6901990

[28] Ye WY, Li J, Li X, Yang XZ, Weng YY, Xiang WW, et al. Predicting the One-Year prognosis and mortality of patients with acute ischemic stroke using red blood cell distribution width before intravenous thrombolysis. Clin Interv Aging. 2020;15:255–63. 32110004 10.2147/CIA.S233701 PMC7039056

[29] Wang L, Wang C, Wu S, Li Y, Guo W, Liu M. Red blood cell distribution width is associated with mortality after acute ischemic stroke: a cohort study and systematic review. Ann Transl Med. 2020;8(4):81. 32175374 10.21037/atm.2019.12.142 PMC7049007

[30] Marta-Enguita J, Navarro-Oviedo M, Rubio-Baines I, Aymerich N, Herrera M, Zandio B, et al. Association of calprotectin with other inflammatory parameters in the prediction of mortality for ischemic stroke. J Neuroinflamm. 2021;18(1):3. 10.1186/s12974-020-02047-1 PMC7786493 33402185

[31] Chamorro A, Meisel A, Planas AM, Urra X, van de Beek D, Veltkamp R. The immunology of acute stroke. Nat Rev Neurol. 2012;8(7):401–10. 22664787 10.1038/nrneurol.2012.98

[32] Bai YD, Yang YR, Mu XP, Lin G, Wang YP, Jin S, et al. Hydrogen Sulfide Alleviates Acute Myocardial Ischemia Injury by Modulating Autophagy and Inflammation Response under Oxidative Stress. Oxid Med Cell Longev. 2018;2018:3402809. 30154948 10.1155/2018/3402809 PMC6093072

[33] Bester J, Swanepoel AC, Windberger U. Editorial: pathological changes in erythrocytes during inflammation and infection. Front Physiol. 2022;13:943114. 35784890 10.3389/fphys.2022.943114 PMC9247548

[34] Yang Y, Liang S, Geng J, Wang Q, Wang P, Cao Y, et al. Development of a nomogram to predict 30-day mortality of patients with sepsis-associated encephalopathy: a retrospective cohort study. J Intensive Care. 2020;8:45. 32637121 10.1186/s40560-020-00459-y PMC7331133

[35] Herlitz-Cifuentes H, Vejar C, Flores A, Jara P, Bustos P, Castro I, et al. Plasma from patients with rheumatoid arthritis reduces nitric oxide synthesis and induces reactive oxygen species in A Cell-Based biosensor. Biosensors-Basel. 2019;9(1):32. 30818887 10.3390/bios9010032 PMC6468433

[36] Yang M, Vousden KH. Serine and one-carbon metabolism in cancer. Nat Rev Cancer. 2016;16(10):650–62. 27634448 10.1038/nrc.2016.81

[37] Liu YH, Chen YH, Ko CH, Kuo CW, Yen CC, Chen W, et al. SOD3 and IL-18 predict the first kidney disease-Related hospitalization or death during the One-Year Follow-Up period in patients with End-Stage renal disease. Antioxidants-Basel. 2022;11(6):1198. 35740095 10.3390/antiox11061198 PMC9231321

[38] Korei C, Szabo B, Varga A, Barath B, Deak A, Vanyolos E, et al. Hematological, Micro-Rheological, and metabolic changes modulated by local ischemic Pre- and Post-Conditioning in rat limb Ischemia-Reperfusion. Metabolites. 2021;11(11):776. 34822434 10.3390/metabo11110776 PMC8625580

[39] Xu W, Huo J, Chen G, Yang K, Huang Z, Peng L, et al. Association between red blood cell distribution width to albumin ratio and prognosis of patients with sepsis: A retrospective cohort study. Front Nutr. 2022;9:1019502. 36211519 10.3389/fnut.2022.1019502 PMC9539557

[40] Kim KM, Lui LY, Cauley JA, Ensrud KE, Orwoll ES, Schousboe JT, et al. Red cell distribution width is a risk factor for hip fracture in elderly men without Anemia. J Bone Min Res. 2020;35(5):869–74. 10.1002/jbmr.3963 PMC7744556 31991005

